# BurkHostGEN: a study protocol for evaluating variations in the *Burkholderia pseudomallei* and host genomes associated with melioidosis infection

**DOI:** 10.12688/wellcomeopenres.19809.1

**Published:** 2023-08-17

**Authors:** Kesorn Angchagun, Phumrapee Boonklang, Chalita Chomkatekaew, Sukritpong Pakdeerat, Gumphol Wongsuwan, Premjit Amornchai, Vanaporn Wuthiekanun, Salwaluk Panapipat, Thatsanun Ngernseng, Naomi Waithira, Steve Walton, Direk Limmathurotsakul, Anoree Surawong, Suwatthiya Siriboon, Parinya Chamnan, Narisara Chantratita, Susie Dunachie, Jukka Corander, Emma E. Davenport, Julian Knight, Julian Parkhill, Sharon J. Peacock, Nicholas R. Thomson, Nicholas P.J. Day, Claire Chewapreecha

**Affiliations:** 1 Mahidol-Oxford Tropical Medicine Research Unit, Faculty of Tropical Medicine, Mahidol University, Salaya, Nakhon Pathom, 10400, Thailand; 2 Wellcome Sanger Institute, Hinxton, England, CB10 1SA, UK; 3 Sunpasitthiprasong Regional Hospital, Ubon Ratchathani, 34000, Thailand; 4 Department of Microbiology and Immunology, Faculty of Tropical Medicine, Mahidol University, Salaya, Nakhon Pathom, 10400, Thailand; 5 Nuffield Department of Medicine, Centre of Tropical Medicine and Global Health, University of Oxford, Oxford, England, OX3 7LG, UK; 6 Institute of Basic Medical Sciences, Faculty of Medcine, University of Oslo, Oslo, Oslo, 0317, Norway; 7 Wellcome Centre for Human Genetics, University of Oxford, Oxford, England, OX3 7BN, UK; 8 Department of Veterinary Medicine, University of Cambridge, Cambridge, England, CB3 0ES, UK; 9 Department of Medicine, University of Cambridge, Cambridge, England, CB2 0SP, UK; 10 Department of Clinical Medicine, Faculty of Tropical Medicine, Mahidol University, Salaya, Nakhon Pathom, 10400, Thailand

**Keywords:** melioidosis, genomics, diagnostics, epidemiology, microbiology, bioresources

## Abstract

**Background:** Melioidosis is a frequently fatal disease caused by an environmental bacterium *Burkholderia pseudomallei*. The disease is prevalent in northeast Thailand, particularly among rice field farmers who are at risk of bacterial exposure through contact with contaminated soil and water. However, not all exposure results in disease, and infection can have different infection outcomes. Our hypothesis is that the acquisition and outcomes of melioidosis may be influenced by genetic factors of the bacterium, the host, or a combination of both. To address this hypothesis, we aim to collect, sequence, and analyse genetic data from melioidosis patients and controls, along with isolates of *B. pseudomallei* obtained from patients. Additionally, we will study the metagenomics of the household water supply for both patients and controls, including the presence of *B. pseudomallei.*

**Methods:** BurkHostGEN is an ongoing observational study being conducted at Sunpasitthiprasong Hospital, Ubon Ratchathani, Thailand. Weare obtaining consent from 600 melioidosis patients and 700 controls, spanning both sexes, to collect 1 mL of blood for host DNA analysis, 3 mL of blood for RNA analysis, as well as 5 L of household water supply for metagenomic analysis. Additionally, we are isolating *B. pseudomallei* from the melioidosis patients to obtain bacterial DNA. This comprehensive approach will allow us to identify *B. pseudomallei* and their paired host genetic factors associated with disease acquisition and severity. Ethical approvals have been obtained for BurkHostGEN. Host and bacterial genetic data will be uploaded to European Genome-Phenome Archive (EGA) and European Nucleotide Archive (ENA), respectively.

**Conclusions:** BurkHostGEN holds the potential to discover bacterial and host genetic factors associated with melioidosis infection and severity of illness. It can also support various study designs, including biomarker validation, disease pathogenesis, and epidemiological analysis not only for melioidosis but also for other infectious diseases.

## Introduction

Melioidosis is a public health burden yet an often neglected tropical disease, causing an estimated 4.64 million disability-adjusted life-years (DALYs), primarily due to years of life lost (YLL) ^1, 2^. The disease is caused by *Burkholderia pseudomallei,* a Gram-negative bacterium found in soil and contaminated water in disease-endemic areas. Infections can occur through inoculation, inhalation, or ingestion of the bacterium, resulting in either subclinical or clinical infection. Fatalities are observed in 10 to 40% of cases ^2^. Epidemiological studies conducted in northeast Thailand, an area endemic for melioidosis, has provided valuable insights into the disease. The reported prevalence of culture-confirmed clinical infection in this region is approximately 8.73 cases per 100,000 population per year ^3^. However, serological studies among healthy population in the same area revealed a much higher rate of exposure, with an estimated 1 in 4,600 antibody-producing exposures resulting in clinical infection ^4^. These findings suggest that not all bacterial exposure leads to melioidosis. Clinical manifestations are commonly observed in individuals aged 45 years and above, particularly those with one or more risk factors ^5, 6^. The primary risk factor is regular unprotected contact with *B. pseudomallei*, which is prevalent among agricultural workers in Southeast Asia and highlights the importance of occupational health campaigns. Another common risk factor is diabetes mellitus, which is present in approximately half of melioidosis cases. The prevalence of diabetes in Thailand has increased over the past decade ^7^, further increasing the population at risk of melioidosis.

Although melioidosis is commonly regarded as an opportunistic infection, this does not preclude the possibility that certain *B. pseudomallei* strains or individuals may possess genetic factors that increase their susceptibility to developing melioidosis or having more severe outcomes. Previous studies have used additive heritability scores (h ^2^) to systematically quantify the proportion of variations in the infection outcomes that can be explained by genetic variations in the pathogens and the hosts. For instance, bacterial genetic factors accounted for 70% and 36.5% of invasiveness potential in *Streptococcus pneumoniae* ^8^ and *Neisseria meningitidis* ^9^, respectively. These findings justified conducting a microbial genome-wide association study (mGWAS) to identify bacterial genetic factors associated with transition from carriage to disease, offering insights into disease pathology. On the host side, h ^2^ estimation and human genome-wide association study (hGWAS) have been reported for sepsis ^10, 11^, as well as specific pathogen infections, such as SARS-CoV-2 ^12^, HIV ^13^, hepatitis ^14^, *Salmonella* infection ^15^, tuberculosis ^16, 17^, and malaria ^18^. Although sample sizes are still limited, joint host-pathogen studies have emerged for invasive pneumococcal disease ^8^, tuberculosis ^19^ and hepatitis C ^20^. Collectively, these genetic studies shed light on the underlying biological mechanisms involved in heterogeneous infectious responses and facilitate the identification of specific genetic markers in pathogens and hosts. This knowledge is pertinent for future advancements in treatment stratification and vaccine development.

Currently, a comprehensive genetic study dedicated to melioidosis, along with a genetic database encompassing both bacterial and host genetic variations are lacking. To address this gap, we established a *Burkholderia pseudomallei* and Host Genetic cohort (BurkHostGEN). This initiative aims to collect and consolidate existing genetic data on both the bacterium and host to identify markers associated with the disease. BurkHostGEN will also create a community database that can be shared among researchers and healthcare professionals. The resulting data and analyses will pave the way for future progress in diagnosis, treatment stratification, and development of vaccines for this challenging infection. The first data collection started in October 2019, with data and analyses expected to be complete in August 2025.

### Ethical approvals: Dissecting the genetic basis of melioidosis infection

The study received ethical approval from the Sunpasitthiprasong Hospital Ethical Review Board (015/62C) and the Oxford Tropical Research Ethics Committee (OxTREC, 25-19). The initial approval from the Sunpasitthiprasong Hospital Ethical Review Board was granted on 7th August 2019, followed by an amendment approval on 23 ^rd^ July 2020. The Oxford Tropical Research Ethics Committee initially approved the protocol on 30 ^th^ May 2019, and subsequently approved an amendment on 7 ^th^ September 2020. The amendment was to include household water sampling activities.

## Protocol

### Objectives and purposes

***Primary objective***

To identify bacterial and patient biomarkers that are associated with melioidosis acquisition and disease outcome.

***Secondary objectives***

- To understand how host diabetic status modulates the effect of the bacterial and patient biomarkers in disease.
- To establish a genetic database of pathogen and host dedicated to melioidosis.

### Study design

**Figure 1.**
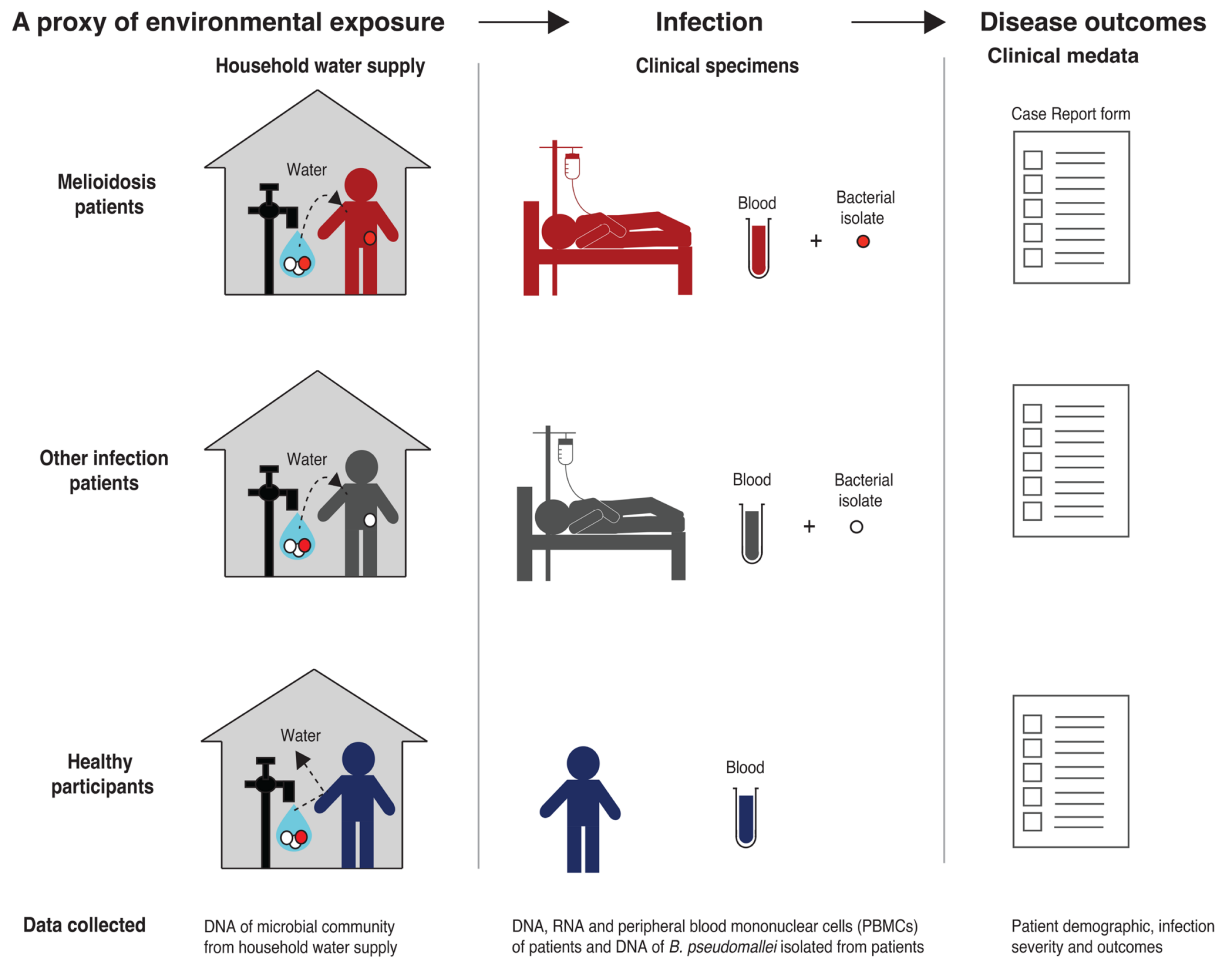
Study design. BurkHostGEN aims to collect, sequence and investigate the genetics of melioidosis patients, the bacterium that cause the disease in patients, as well as the bacteria recovered from patient’s household water supply.

This is an observational study that collects and analyses linked host and bacterial genetics samples ( Figure 1). Firstly, DNA and RNA blood samples are collected from both melioidosis patients and controls to explore the host factors contributing to melioidosis susceptibility. Secondly, DNA from *B. pseudomallei* isolated from respective melioidosis patients is collected to investigate the bacterial factors involved in disease acquisition and infection outcomes. Thirdly, DNA of the microbiome of the household water supply of both melioidosis patients and controls is collected to examine their environmental exposure. Additionally, demographic data including age, self-reported sex and ancestry, alongside clinical data linked to these samples are collected to provide context for genetic findings.

To ensure statistical power as well as budget and timeline feasibility, we plan to recruit a sample size of 600 melioidosis patients, 550 healthy controls and 150 patients with other infections as additional controls. Considering that 50% of melioidosis patients have diabetes mellitus, we recruited individuals with and without diabetes mellitus in equal proportions for the control groups to match the melioidosis group. BurkHostGEN Case Report Forms ^21^, Patient information sheet (PIS) and informed consent forms (ICFs) ^22^ can be found as *Extended data*.

### Study site and population

The study is being conducted at Sunpasitthiprasong Hospital, a regional hospital located in Ubon Ratchathani, Thailand. The hospital serves as a vital healthcare provider for the local community and surrounding areas and has gained recognition for its expertise in diagnosing and treating melioidosis, given the high incidence of disease in the region. For the study, the melioidosis, and other infection groups are being recruited from the infectious department of the hospital. The healthy group are being sourced from two different hospital departments. Healthy controls without diabetes mellitus are being recruited from the hospital blood donor clinic at the hospital. Healthy controls with diabetes are being recruited from diabetes outpatient clinic. During the recruitment process, effort is being made to ensure representation across all age groups and both sexes.

### Participant inclusion criteria

***Melioidosis group***

- Age ≥ 18 years old.
- Culture-confirmed infection of *B. pseudomallei* from any clinical samples.
- Willingness to participate in the study and written, informed consent obtained from patient or their relative.
- Resident in northeast Thailand for at least the previous two years.

***Healthy group***

- Age ≥ 18 years old.
- Currently well (non-diabetic) or outpatient of diabetic clinic (diabetic group) but have no other medical problems requiring hospital supervision.
- Willingness to participate in the study and written, informed consent obtained from patient or their relative.
- Resident in northeast Thailand for at least the previous two years.

***Other infection group***

- Age ≥ 18 years old.
- Culture-confirmed infection by bacterial pathogens other than *B. pseudomallei*.
- Willingness to participate in the study and written, informed consent obtained from patient or their relative.
- Resident in northeast Thailand for at least the previous two years.

### Participant exclusion criteria

***Melioidosis group***

- Current tuberculosis (TB) or treatment of TB within the last six months.
- Documented human immunodeficiency virus (HIV) infection, chemotherapy, or other immunosuppressant therapy in the last 12 months.
- Pregnancy.

***Healthy group***

- Previous history of melioidosis.
- Significant acute intercurrent illness.
- Current TB or treatment of TB within the last six months.
- Documented HIV infection, chemotherapy, or other immunosuppressant therapy in the last 12 months.
- Pregnancy.

***Other infection group***

- Previous history of melioidosis.
- Significant acute intercurrent illness.
- Current TB or treatment of TB within the last six months.
- Documented HIV infection, chemotherapy, or other immunosuppressant therapy in the last 12 months.
- Pregnancy.

### Participant recruitment and sample collection

***Screening and enrolment***

Upon receiving a culture-confirmed diagnosis, eligible patients at Sunpasitthiprasong Hospital are approached by a member of study staff. A screening process based on participant inclusion criteria is conducted. If the patients meet the criteria, they are presented with a written participant information (PIS) and an informed consent form (ICF) in Thai language. In cases where the patient lacks capacity, the information is provided to their relative.

The participants receive a clear explanation of the study, including its nature, protocol implications, known side effects and any risks. They are explicitly informed of their right to withdraw from the study at any time, without prejudice for future care and without the need to provide a reason for withdrawal. Sufficient time, at least 10 minutes are given for consideration, along with an opportunity to ask questions to the investigator or other independent parties.

Written informed consent is obtained through dated signatures of the participant or relative, as well as the person presenting and obtaining consent. In cases where the participant or relative is illiterate, a thumbprint is obtained. A copy of the signed informed consent forms is provided to the participant or responsible relative, while the original signed forms are retained at the study site.

After the participant has been enrolled into the study, they are assigned a unique study number. These numbers consist of a letter indicating the group they belong to, with “M”, “H” and “I” representing melioidosis, healthy, and other infection groups, respectively.

***Sample collection***

For each participant, two to three types of samples are collected to support the study objectives.

Blood samples ( Table 1) are collected once at enrolment for all studied groups. Participants are informed about the blood drawing procedure, and preferred sites for phlebotomy are the median antecubital and basilica veins in the upper extremity. Alternative sites such as veins on the dorsum on the hand and other forearm veins are used if necessary. The collected blood is used for DNA and RNA profiling, addressing the primary study objective. Additionally, glycated haemoglobin (HbA1c) levels are measured to assess the impact of chronic hyperglycaemia on infection across all groups. Patient peripheral blood mononuclear cells (PBMC) are also collected to serve as cell models for experimental validation.

**Table 1.** Different types and amounts of samples collected are tabulated below.

Studied group	Ranges of specimens from host blood sample					DNA of clinical bacterial sample	DNA of environmental microbial community
	DNA	RNA	HbA1c	PBMC *	total		
Melioidosis	1 mL	3 mL	1 mL	12 mL	17 mL	A single colony of *B. pseudomallei*	A plate-sweep culture from concentrated 5 L of water
Other infection	1 mL	3 mL	1 mL	12 mL	17 mL	N/A	A plate-sweep culture from concentrated 5 L of water
Healthy individual	1 mL	3 mL	1 mL	12 mL	17 mL	N/A	A plate-sweep culture from concentrated 5 L of water

*If participant in melioidosis and other infection groups are very ill and attending physician rules out that > 5 mL of blood withdrawal could impact the condition, PBMC collection may be omitted. HbA1c, haemoglobin; PBMC, peripheral blood mononuclear cells; N/A, not applicable.

A single colony pick of *B. pseudomallei,* cultured from the specimens of respective melioidosis patients, is obtained from the Sunpasitthiprasong hospital Central Lab, where routine bacterial cultures are performed for diagnostic purposes.

The water supply samples from both melioidosis patients and controls are collected within three months after participant enrolment in the project, with efforts made to minimise seasonal fluctuations.

### Sample processing

***Host blood samples***

Blood samples are collected and stored at -80°C in Ubon Ratchathani until the recruitment phase is completed. EDTA tubes, and Tempus™ RNA blood tube are used to preserve blood DNA and RNA, respectively. The DNA extraction from whole blood will be performed using QIAamp® DNA blood kits. The extracted DNA will then undergo whole genome sequencing to achieve 15x coverage using Novaseq with PE 150 bp read length. Similarly, RNA extraction will be performed from whole blood using Tempus™ Spin RNA isolation kit. The RNA samples will undergo globin depletion and will be sequenced using Novaseq with PE 150 bp read length.

***Bacterium isolated from melioidosis patients***

*B. pseudomallei* isolated from melioidosis patients recruited for this study are stored at -80°C in Ubon Ratchathani. Subsequently, they will be transported in batches to Bangkok for DNA extraction under biosafety level 3 (BSL3) laboratory. The DNA extraction will be performed using QIAamp® DNA mini kit, followed by sequencing using Novaseq with 150 bp read length.

***Bacterial community recovered from host household water supply***

Water samples collected from the household supply of all study participants are processed according to described methodology ^6^. To facilitate the growth of *B. pseudomallei* while inhibiting fungal growth, selective plates are used. Following growth on selective plates, all microbes are plate-swept for further investigation. Additionally, single-colony picks of visible *B. pseudomallei*, along with plate-sweep microbes are stored at -80°C in Ubon Ratchathani before batch transportation to Bangkok. DNA extraction for plate-sweep microbial community will be performed using QIAGEN Genomic tips, which minimise DNA shearing to facilitate long-read sequencing. The microbial DNA will then undergo sequencing using GridION with ligation library preparation on a pool of up to 10 samples per flow cell. As for *B. pseudomallei* single colony picks from the environment, they will be sequenced using Novaseq with the same protocol used for *B. pseudomallei* isolated from patients.

### Data analysis and statistical consideration

***Identification of differential host gene expression signatures based on infection severity***

Our hypothesis is that gene expression signatures differ between individuals with different infection severity. We will categorise the groups as follows: a healthy population with no or subclinical symptoms, a group with mild melioidosis infection that recover, and a group with severe infection resulting in mortality within 28 days. To identify genes that vary among and between these groups, we will perform multi-group differential expression analysis using ANOVA while controlling for common comorbidities and covariates such as participant age and sex. We will also test for consistency with the previously defined sepsis response signature (SRS) ^23, 24^. The SRS1, SRS2, and SRS3 profiles will define patients with an immunosuppressed profile, immunocompetent profile, and healthy individuals, respectively. We will compare the signatures obtained from melioidosis patients to those obtained from the other infection group to determine if the identified signatures are unique to melioidosis infection.

***Exploring host regulatory markers affecting gene expression in melioidosis***

We hypothesise that part of the variation in gene expression is influenced by regulatory genetic determinants. To investigate this, we will perform expression quantitative trait loci (eQTL) analysis. Using a linear model, we will test the correlation between each single nucleotide polymorphism (SNP) and the nearby gene expression. Principal components that define the population structure will be included as covariates. We will search for enrichment of transcription factor binding motifs within these expression-associated SNPs to identify potential regulatory markers affecting gene expression.

***Examining the impact of host diabetic status or anti-diabetic treatment on differential host gene expression***

While individuals with diabetes have an increased risk of developing melioidosis, they have reduced risk of mortality compared to non-diabetic patients ^25^. We aim to investigate how the diabetic status of patients may modulate the outcomes in melioidosis. For each gene showing differential expression, we will compare two nested logistic regression models: one assessing the association with outcomes alone, and the other including host diabetic status and an interaction terms. By doing so, we will identify differentially expressed genes whose effects may be influenced, either dampened or enhanced, by the host’s diabetic status or anti-diabetic treatment.

***Bacterial factors influencing disease severity***

Our goal is to examine whether specific genetic variants in *B. pseudomallei* influence the outcomes of melioidosis. Using a genome-wide association study (GWAS), we will identify associations between bacterial genetic variants (genes, unitigs, and core SNPs) and clinical phenotypes, including 28-day mortality, SOFA scores, and patient diabetic status. To account for *B. pseudomallei* population structure, a phylogeny and principal component analysis (PCA) defining the bacterial population structure will be used as covariates in the GWAS. The expected outcome will be a list of *B. pseudomallei* variants associated with melioidosis outcomes. Additionally, we will provide a summary statistic to facilitate future meta-analysis in *B. pseudomallei* research.

***Impact of host diabetic status or anti-diabetic treatment on bacterial factors***

We also hypothesise that the severity of melioidosis, influenced by bacterial genetic variants, may be modulated by the host’s diabetic status and/or anti-diabetic treatment. To explore this hypothesis, we will compare two nested models: one assessing the association with outcomes alone, and the other incorporating host diabetic status and an interaction terms. The findings are expected to yield a catalogue of *B. pseudomallei* disease-severity variants, highlighting their potential influence, whether attenuated or amplified, by the host’s diabetic status or anti-diabetic treatment.

***Diversity of B. pseudomallei in the household water supply of melioidosis and control groups***

To better understand the transition from environmental *B. pseudomallei* to infection, we proposed to compare genome assemblies of *B. pseudomallei* detected in patient’s household water supply to those causing disease in the patients. Previous studies relied on culture-based approaches to identify *B. pseudomallei*. Due to its relatively slow growth, other microbes in the environment may outcompete *B. pseudomallei*, resulting in invisible colonies and negative culture results. To overcome this limitation, we will employ a new plate-sweep followed by sequencing technique to allow us to capture previously undetected *B. pseudomallei* population.

### Sample size consideration

***Analysis on host differential gene expression***

**Figure 2.**
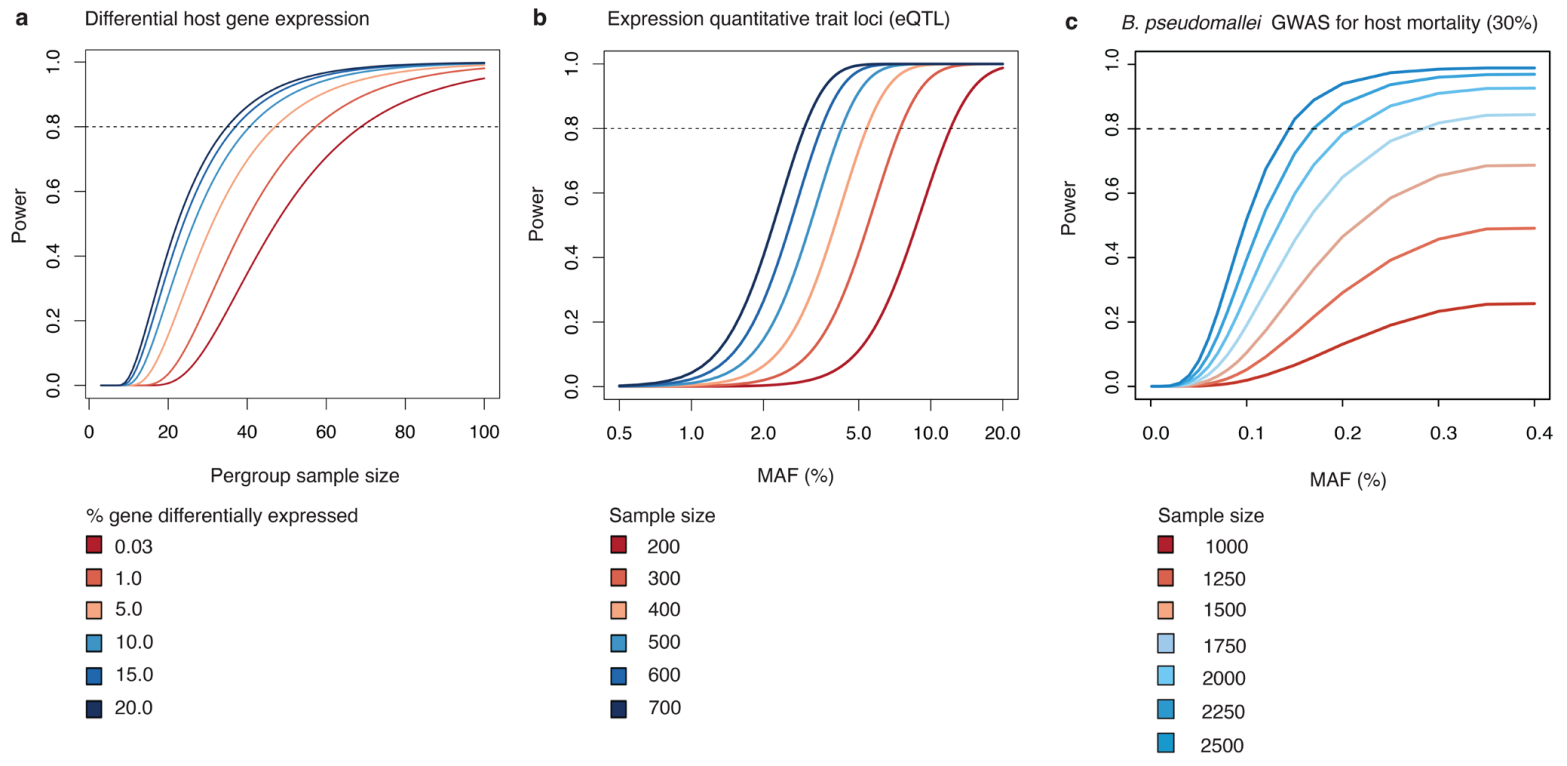
Power calculation. ( **a**) Sample size required to detect host differential host gene expression. ( **b**) sample size required for eQTL analysis. ( **c**) sample size required for bacterial GWAS for host 28-day mortality. eQTL, expression quantitative trait loci; GWAS, genome-wide association study; MAF, minor allele frequencies.

For two sample RNA-seq experiment design, we used a linear model ^26^ to estimate sample size with predicted proportion of differentially expressed genes for each possible condition (healthy *vs.* recovered melioidosis, recovered melioidosis *vs.* fatal melioidosis, and healthy *vs.* fatal melioidosis). Assuming that all genes have homogenous read counts, we estimated that a group size of 41 is needed to detect a 1.5-fold change in gene expression, with false discovery rate (FDR) at 0.05 with a statistical power of 80%. With an estimated melioidosis mortality rate at 30%, a group size of 550 healthy individuals, 420 recovered melioidosis cases, and 180 fatal melioidosis cases which could be achieved in the study time frame should allow sufficient statistical power ( Figure 2a).

***Analysis on host expression quantitative loci (eQTL)***

Given a SNP and a continuous gene expression data, we used a linear regression ^27^ to calculate the sample size with different minor allele frequencies (MAF). Using Bonferroni correction for 200,000 hypotheses based on 20,000 genes and an average of 10 SNPs in proximity of each gene as in 28, we estimated that the size of 600 samples, which is the feasible size collected through this project and compiled with previous reports ^29, 30^, will give 80% power for detecting eQTL with a MAF at 0.03 ( Figure 2b).

***Analysis of bacterial genetic variants***

Given different units of genetic variations (gene presence, unitig, and SNP), and a binary disease outcomes (estimated 30% mortality), we used an additive model ^31^ to calculate the sample size with different MAF. Using a genome-wide significant cut-off at 5 × 10 ^-8^ and genotype relative risk of 1.5, our combined size of over 2,400 *B. pseudomallei* genomes collected in the same endemic area under this study and those reported in 6, 29, 32 has sufficient power to detect associations with MAF of 0.15 ( Figure 2c).

Considering the exploratory nature of the remaining objectives, the sample size was not based on power calculations.

## Ethics

This study is conducted in compliance with the conditions stipulated by the sponsor and local Ethics Committee/Institutional Review Board (EC/IRB), as well as applicable regulatory requirements and ICH Good Clinical Practice (GCP) guidelines.

## Risks

This study is categorised as minimal risk since it does not involve experimental interventions or investigational new drugs. The total amount of blood collected from patients (up to 17 mL) is considered safe. Stored samples are used for diagnostic purposes relevant to the study aims, and consent is obtained from patients for the storage of all clinical specimens.

## Participant confidentiality

All study-related information is securely stored at the study site. Subject information is kept in locked file cabinets, with restricted access limited to study staff. The data of participants’ home addresses are used for the one-off collection of water sample and are strictly limited to the water collection team. Specimens, reports, data collection forms, and administrative records are identified by anonymised codes. Personal identifiers such as names are stored separately from study records. The study database has password-protected access systems. Subject information will not be released without written permission. Research dataset will be pseudonymised. This allows for eventualities such as feedback of individual data or withdrawal of consent. Subject names or identities will not be disclosed. The risk of re-identification from the published data is considered very low.

## Data handling and record keeping

Clinical data recorded on Case Report Forms (CRF) are entered into the MACRO EDC, a GCP-compliant data management system. The database is password-protected and incorporates internal quality checks to ensure data consistency, completeness, and accuracy. Study participants are identified by unique participant numbers in the database.

Participant records are stored in binders or scanned and stored electronically. Given participant consent, anonymised data and results from blood and water sample analyses stored in the database maybe shared with other researchers. Personal information is anonymised to protect privacy.

## Genetic data, reuse of data and data sharing

The project’s outputs, including sequencing data and associated metadata hold value for other researchers. To ensure reproducibility and comply with the Wellcome Trust policy, the sequenced data will be stored in two separate archives. The European Genome-Phenome Archive (EGA), a managed access database, will host the genomic and transcriptomic data of human participants. The bacterial genomic data will be stored in the European Nucleotide Archive (ENA), an open access database.

To comply with the General Data Protection regulation, pseudonymised participant data and microbiological data will be shared with researchers. To safeguard participant privacy, stringent measures will be implemented, such as the removal of rare genetic variants or traits unique to only a few individuals, ensuring that the study participants cannot be identified from any shared data. This approach effectively maintains participant confidentiality and privacy while enabling the dissemination and utilisation of research data among the scientific community. Access to human datasets will be carefully managed and granted transparently to appropriately qualified researchers.

Anonymised participant data submitted to EGA for data reuse will include sex, phenotype (disease and diabetic status), self-reported ancestry, and a study number. Additionally, the summary statistics to be released as part of the future publication will encompass variant identifiers (rsID, chromosome position), effect alleles, effect size, confident intervals, and associated statistics. The risk of re-identification of individual research participants, even if unlikely, is considered low.

## Study status

Ethical approvals were granted for BurkHostGEN in August 2019 and recruitment began shortly afterwards. To date, over 1,000 participants have been successfully enrolled. The collected samples are scheduled to undergo processing, sequencing and analysis with an expected completion in August 2025.

A portion of the collected data has been reported in a preprint and can be found on medRxiv (doi: https://doi.org/10.1101/2023.05.06.23289616). The preprint relies on BurkHostGEN clinical data collected between October 2019 to December 2022. The dataset provides a rationale for the development of a rapid CRISPR diagnostic test. This prompted us to develop the rapid CRISPR-based diagnosis test reported in the preprint. Currently, the preprint is undergoing revisions within a peer-reviewed journal.

## Conclusions

BurkHostGEN aims to link environmental metagenomic, pathogen genomic and host genomic in a less studied population of northeast Thailand. This approach will provide unique insights into the patients’ environmental exposures, the infecting pathogen, *B. pseudomallei*, and the patient population. The genetic and metadata from BurkHostGEN can facilitate the discovery and validation of genetic factors associated with melioidosis development and outcomes in both the pathogen and the hosts. This comprehensive data allows for an initial exploration of the interplay between *B. pseudomallei* and host genetics, potentially revealing genetic factors associated with melioidosis development and outcomes. Given the current sample size, the initial results may be confined to common alleles in the pathogen and regulatory alleles in the hosts. However, there are plans for future data expansion to capture rarer alleles. This dataset holds many potentials, enabling genetic investigations not only specific to melioidosis but also to other common infectious diseases prevalent in this less studied global region.

## Data Availability

No data are associated with this article. Figshare: BurkHostGEN Case Report Forms (CRF). https://doi.org/10.6084/m9.figshare.23908641 ^21^ This project contains the following extended data: BurkHostGEN_CRF_Melioid_V5.0_12May20_approved .doc BurkHostGEN_CRF_Healthy_V.5.0_12May20_approved.doc BurkHostGEN_CRF_Other-Infection_V5.0_12May20_approved.doc Figshare: BurkHostGEN Patient information sheet (PIS) and informed consent forms (ICF). https://doi.org/10.6084/m9.figshare.23905611 ^22^ This project contains the following extended data: BurHostGEN_PIS-ICF_Healthy_V.5.1_22July2020_approved.docx BurHostGEN_PIS-ICF_Melioid_V.5.1_22July2020_approved.docx BurHostGEN_PIS-ICF_Other-Infection_V.5.1_22July2020_approved.docx Data are available under the terms of the Creative Commons Attribution 4.0 International license (CC-BY 4.0).
