# Supplementary material for: BurkHostGEN: a study protocol for evaluating variations in the Burkholderia pseudomallei and host genomes associated with melioidosis infection
Source: Wellcome Open Res. 2023 Nov 2;8:347. Originally published 2023 Aug 17. [Version 2] doi: 10.12688/wellcomeopenres.19809.2 (PMC10624953; doi:10.12688/wellcomeopenres.19809.2)
Supplement: Peer review history

# Reviewer report: Reviewer response for version 2

**DOI:** 10.21956/wellcomeopenres.22585.r69547

**Published:** 2023-11-03

**Authors:** Sheila Nathan

**Affiliations:** 1 Faculty of Science and Technology, School of Biosciences and Biotechnology, National University of Malaysia, Bangi, Selangor, Malaysia

The authors have addressed the concerns I raised in my initial peer review report appropriately and I am happy to recommend indexing with no further comments or changes.

Is the study design appropriate for the research question?

Yes

Is the rationale for, and objectives of, the study clearly described?

Partly

Are sufficient details of the methods provided to allow replication by others?

No

Are the datasets clearly presented in a useable and accessible format?

Not applicable

Reviewer Expertise:

Molecular microbiology; host-pathogen interaction; Burkholderia pseudomallei genetics

I confirm that I have read this submission and believe that I have an appropriate level of expertise to confirm that it is of an acceptable scientific standard.

# Reviewer report: Reviewer response for version 1

**DOI:** 10.21956/wellcomeopenres.21941.r65464

**Published:** 2023-10-12

**Authors:** Chiranjay Mukhopadhyay, Ranita Ghosh Dastidar

**Affiliations:** 1 Department of Microbiology, Kasturba Medical College and Hospital, Manipal Academy of Higher Education, Manipal, Karnataka, India; 2 Department of Biochemistry, Kasturba Medical College, Manipal, Karnataka, India

In the study protocol entitled “BurkHostGEN: a study protocol for evaluating variations in the *Burkholderia* *pseudomallei* and host genomes associated with melioidosis infection” by Kesorn Angchagun *et al.*, the authors have emphasized that not all bacterial exposures lead to melioidosis. Acquisition and further the outcomes of melioidosis vary largely due to genetic factors of both the acquired bacteria and the genetic makeup of the individual. With this fact in focus, the authors have undertaken an initiative to perform a detailed genomic study hypothesizing that genetic factors of both bacteria and host (separately or combinatorially) determine the acquisition and outcome patterns of patients. The protocol includes comparative DNA and RNA level studies from the patient and control blood samples as well as comparing their HbA1c levels. The protocol also enables comparison of DNA from bacteria collected from patient sample and community water source in nearby areas of patients and controls by metagenomic analyses. It also collects and stores the patient PBMCs for future utilization as cell models. The sample collection procedure is already over but can continue further as well. The sequencing and analysis, when complete, will provide the opportunity to discover several novel bacterial and host genetic factors and also can be followed by all melioidosis-prone south eastern Asia and northern Australian regions and beyond for better understanding, prevention and cure of the disease. This study protocol can further serve as basic guideline for the study of many other infectious diseases and emerging tropical diseases and also help generate community database nationally and globally.

 After careful review of the manuscript, these reviewers feel that there are following minor concerns regarding the manuscript, which after being addressed can allow the approval of this protocol for indexing.

1. For DNA extraction of environmental microbial community, emphasis has been on the plate-sweep culture from concentrated 5 litre of water (as shown in table 1). The disadvantages of single-colony pick method should be elaborated if any. Methods section, however, mentions the use of both plate-sweep  and single-colony pick methods being used for sequencing using GridION (with ligation library preparation on a pool of up to 10 samples per flow cell) and Novaseq (as for patient sample isolation) respectively. Please clarify.
2. In section “Diversity of *B. pseudomallei* in the household water supply of melioidosis and control group”, it is mentioned that slow growth of *B. pseudomallei* can lead to overgrowth of other microbes and hence negative culture results due to invisible colonies. This section can be better aligned with the methods section for better clarification.
3. Please mention clearly - Are all sequencing procedures done under BSL3 laboratory facilities or only DNA/RNA extraction methods?
4. In data analysis section, one of the infection severity groups includes, “a healthy population with no or subclinical symptoms” – please elaborate subclinical symptoms.
5. The definition for ‘mild melioidosis’ may be more clear.
6. Since a portion of the collected data has been reported in another preprint awaiting peer-review, assure that this does not create any conflict of interest amongst participating individuals or groups. A statement to that effect will be preferred that states these two groups are identical or have no conflicts.
7. In sample collection section, preferred and alternative sites of phlebotomy are mentioned. Any information related to any expected differences in the HbA1c reports and amount of PBMC that can be collected that can vary based on the site of blood collection (for phlebotomy) if any should be mentioned (as a matter of fact if known or as an assumption).

Is the study design appropriate for the research question?

Yes

Is the rationale for, and objectives of, the study clearly described?

Yes

Are sufficient details of the methods provided to allow replication by others?

Yes

Are the datasets clearly presented in a useable and accessible format?

Yes

Reviewer Expertise:

Infectious Diseases, Emerging Tropical Diseases, Melioidosis

We confirm that we have read this submission and believe that we have an appropriate level of expertise to confirm that it is of an acceptable scientific standard.

# Reviewer report: Reviewer response for version 1

**DOI:** 10.21956/wellcomeopenres.21941.r65463

**Published:** 2023-09-04

**Authors:** Sheila Nathan

**Affiliations:** 1 Faculty of Science and Technology, School of Biosciences and Biotechnology, National University of Malaysia, Bangi, Selangor, Malaysia

This study is proposed to identify *Burkholderia pseudomallei* and host genetic factors that influence the outcome of an infection by this tropical bacterium. Additional factors included in this study are the risk factors of diabetes and environmental exposure to the pathogen. Overall, this is an ambitious but timely research undertaking, which expects to contribute new knowledge to benefit the design of diagnostic biomarkers as well potentially identify susceptibility markers to forecast disease severity.

 The standard of the English used requires improvement in various sections. Some examples within the Abstract and Introduction are noted below:

Abstract

- 'infection can have different infection outcomes' - the word "infection" appears twice in the same sentence.
- 'acquisition and outcomes of melioidosis' - one does not acquire the disease.
- 'Weare obtaining' - should be "We are"

Intro

- 'amendment approval' - amended approval
- The use of past-, present- and future tense should be standardised.

General Comments

 "The resulting data and analyses will pave the way for future progress in diagnosis, treatment stratification, and development of vaccines for this challenging infection." - what are the issues or bottlenecks currently faced?

 What was the need to collect and analyse the metagenomic content of household water collected from patients' houses? What is the source of the water supply - treated or untreated? Why is the assumption made that a potential infection route is the consumed water and not from agriculture-based activities (soil sampling)?

"Primary objective - To identify bacterial and **patient **biomarkers that are associated with melioidosis acquisition and disease outcome." Does the study include those individuals who are antibody-positive but do not display symptoms? In this case, they would not be classified as "patients".

 There are a large number of methods to be undertaken in this study. Nonetheless, the completeness of the protocols is lacking (subject to Wellcome Open Research's requirements for describing an ongoing study).

 The blood samples (1400) will be stored until recruitment is completed. When is recruitment expected to be done? How long will it take to perform the downstream experiments and analysis? Do you expect any differences in samples stored for 12 months compared to samples obtained 1 month prior to the completion of recruitment?

*"B. pseudomallei *single colony picks from the environment" - no details related to the selected "environments" are provided as well as on how sampling will be done. If "environment" refers to the household water samples, I suggest retaining the use of a single term rather than inter-changing. Nonetheless, I also refer to my earlier comment on the source of the water - is it ground water in a well or is it treated / untreated pipe/tap water?

 Risks - what are the containment measures to be taken when transporting the bacteria to Bangkok? Will the blood samples taken from melioidosis-confirmed patients be routinely screened for other infectious agents that might compromise the PBMC analysis?

Genetic Data, reuse of data and data sharing - last sentence "The risk of re-identification of individual research participants, even if **unlikely**, is considered low." - I think it should be "likely" instead of "unlikely".

Is the study design appropriate for the research question?

Yes

Is the rationale for, and objectives of, the study clearly described?

Partly

Are sufficient details of the methods provided to allow replication by others?

No

Are the datasets clearly presented in a useable and accessible format?

Not applicable

Reviewer Expertise:

Molecular microbiology; host-pathogen interaction; Burkholderia pseudomallei genetics

I confirm that I have read this submission and believe that I have an appropriate level of expertise to confirm that it is of an acceptable scientific standard, however I have significant reservations, as outlined above.
